# Supplementary material for: The Effect of Consumption of Animal Milk Compared to Infant Formula for Non-Breastfed/Mixed-Fed Infants 6–11 Months of Age: A Systematic Review and Meta-Analysis
Source: Nutrients. 2022 Jan 23;14(3):488. doi: 10.3390/nu14030488 (PMC8838240; doi:10.3390/nu14030488)
Supplement: Supplementary file 1 [file nutrients-14-00488-s001.zip › nutrients-1553181-supplementary.pdf]

Supplementary Document:

The effect of consumption of animal milk compared to infant formula for non-breastfed/mixed-fed infants 6-11 months of age. A systematic review and meta-analysis

Julie M Ehrlich, Joseph Catania, Muizz Zaman, Emily Tanner Smith, Abigail Smith, Olivia Tsistinas, Zulfiqar Ahmed Bhutta, and Aamer Imdad

Table of Content:

**Headings**	**Page Number**
**Text S1:** Search Strategy for electronic data bases	2
**Table S1:** List of publications of included studies	5
**Figure S1:** Effect of animal's milk vs formula milk intake in infants 6-11 month of age on Anemia: Subgroup analysis based on age of initiation	6
**Figure S2:** Effect of animal's milk vs formula milk intake in infants 6-11 month of age weight for age: Subgroup analysis based on age of initiation	6
**Table S2:** List of excluded studies and reason for exclusion	7
**Table S3:** Handling of data from individual studies for inclusion in the meta-analysis	9
**Table S4:** Study definitions of outcome parameters of anemia and gastrointestinal blood loss	9
**References**	10

Text S1: Search Strategy for electronic data bases

Complementary Feeding Strategies

PubMed

((("cow milk*"[tiab] OR "cow's milk*"[tiab] OR "cows milk*"[tiab] OR "bovine milk*"[tiab] OR "bovine’s milk*"[tiab] OR "goat milk*"[tiab] OR "goat's milk*"[tiab] OR "goats milk*"[tiab] OR "caprine milk*"[tiab] OR "buffalo milk*"[tiab] OR "buffalo's milk*"[tiab] OR "camel milk*"[tiab] OR "camel’s milk*"[tiab]OR "camels milk*"[tiab] OR "sheep milk*"[tiab] OR "sheep’s milk*"[tiab] OR "ewe milk*"[tiab] OR "ewe’s milk*"[tiab] OR "ewes milk*"[tiab] OR "ovine milk*"[tiab]) AND ("Infant Formula"[Mesh] OR formula*[tiab])) AND ("Infant"[Mesh] OR infant*[tiab] OR infancy[tiab] OR baby[tiab] OR babies[tiab] OR neonat*[tiab] OR "neo nat*"[tiab] OR newborn*[tiab] OR "new born*"[tiab] OR "newly born*"[tiab] OR "Child"[Mesh] OR child*[tiab] OR youth[tiab] OR juvenile*[tiab])) NOT ("Animals"[Mesh] NOT ("Animals"[Mesh] AND "Humans"[Mesh]))

CINAHL

(TI ("cow milk*" OR "cow's milk*" OR "cows milk*" OR "bovine milk*" OR "bovine's milk*" OR "goat milk*" OR "goat's milk*" OR "goats milk*" OR "caprine milk*" OR "buffalo milk*" OR "buffalo's milk*" OR "camel milk*" OR "camel’s milk*" OR "camels milk*" OR "sheep milk*" OR "sheep’s milk*" OR "ewe milk*" OR "ewe’s milk*" OR "ewes milk*" OR "ovine milk*") OR AB ("cow milk*" OR "cow's milk*" OR "cows milk*" OR "bovine milk*" OR "bovine's milk*" OR "goat milk*" OR "goat's milk*" OR "goats milk*" OR "caprine milk*" OR "buffalo milk*" OR "buffalo's milk*" OR "camel milk*" OR "camel’s milk*" OR "camels milk*" OR "sheep milk*" OR "sheep’s milk*"OR "ewe milk*" OR "ewe’s milk*" OR "ewes milk*" OR "ovine milk*"))
AND
((MH "Infant Formula") OR (TI formula* OR AB formula*))
AND
(((MH "Child") OR (MH "Infant+")) OR (TI (infant* OR infancy OR baby OR babies OR neonat* OR "neo nat*" OR newborn* OR "new born*" OR "newly born*" OR child* OR youth OR juvenile*) OR AB (infant* OR infancy OR baby OR babies OR neonat* OR "neo nat*" OR newborn* OR "new born*" OR "newly born*" OR child* OR youth OR juvenile*)))
NOT
(((MH "Animals+") OR (MH "Animal Studies") OR (TI "animal model*")) NOT (MH "human"))

Scopus

((TITLE-ABS ("cow milk*" OR "cows milk*" OR "bovine milk*" OR "bovine's milk*" OR "goat milk*" OR "goats milk*" OR "caprine milk*" OR "buffalo milk*" OR "buffalo's milk*" OR "camel milk*" OR "camels milk*" OR "sheep milk*" OR "sheep's milk*" OR "ewe milk*" OR "ewes milk*" OR "ovine milk*")) AND (TITLE-ABS (formula*)) AND (TITLE-ABS (infant* OR infancy OR baby OR babies OR neonat* OR "neonat*" OR newborn* OR "new born*" OR "newly born*" OR child* OR youth OR juvenile*)) AND NOT INDEX (medline))

Embase

1 'cow milk'/exp OR 'goat milk'/exp OR 'buffalo milk'/exp OR 'camel milk'/exp OR 'sheep milk'/exp

2 'cow milk*':ti,ab OR 'cows milk*':ti,ab OR 'cow s milk*':ti,ab OR 'bovine milk*':ti,ab OR 'bovine s milk*':ti,ab OR 'goat milk*':ti,ab OR 'goat s milk*':ti,ab OR 'goats milk*':ti,ab OR 'caprine milk*':ti,ab OR 'buffalo milk*':ti,ab OR 'buffalo s milk*':ti,ab OR 'camel milk*':ti,ab OR 'camel s milk*':ti,ab OR 'camels milk*':ti,ab OR 'sheep milk*':ti,ab OR 'sheep s milk*':ti,ab OR 'ewe milk*':ti,ab OR 'ewes milk*':ti,ab OR 'ewe s milk*':ti,ab OR 'ovine milk*':ti,ab

3 #1 OR #2

4 'artificial milk'/exp

5 formula*:ti,ab

6 #4 OR #5

7 'infant'/exp OR 'infancy'/exp OR 'baby'/exp OR 'newborn'/exp OR 'child'/exp OR 'juvenile'/exp

8 infant*:ti,ab OR infancy:ti,ab OR baby:ti,ab OR babies:ti,ab OR neonat*:ti,ab OR 'neo nat*':ti,ab OR newborn*:ti,ab OR 'new born*':ti,ab OR 'newly born*':ti,ab OR child*:ti,ab OR youth:ti,ab OR juvenile*:ti,ab

9 #7 OR #8
10 #3 AND #6 AND #9
11 #10 NOT ([animals]/lim NOT [humans]/lim)

Cochrane CENTRAL
1 cow* NEXT milk*:ti,ab OR bovine* NEXT milk*:ti,ab OR goat* NEXT milk*:ti,ab OR caprine NEXT milk*:ti,ab OR buffalo* NEXT milk*:ti,ab OR camel* NEXT milk*:ti,ab OR sheep* NEXT milk*:ti,ab OR ewe* NEXT milk*:ti,ab OR ovine NEXT milk*:ti,ab
2 MeSH descriptor: [Infant Formula] explode all trees
3 formula*:ti,ab
4 #2 OR #3
5 MeSH descriptor: [Child] explode all trees
6 MeSH descriptor: [Infant] explode all trees
7 infant*:ti,ab OR infancy:ti,ab OR baby:ti,ab OR babies:ti,ab OR neonat*:ti,ab OR neo NEXT nat*:ti,ab OR newborn*:ti,ab OR new NEXT born*:ti,ab OR newly NEXT born:ti,ab OR child*:ti,ab OR youth:ti,ab OR juvenile*:ti,ab
8 #5 OR #6 OR #7
9 MeSH descriptor: [Animals] explode all treMeSH descriptor: [Infant] explode all trees
10 MeSH descriptor: [Humans] explode all trees
11 (#9 NOT(#9 AND #10))
12 #1 AND #4 AND #8
13 #12 NOT #11
14 "accession number" near pubmed
15 #13 NOT #14

Web of Science: 89 results
1 TI=(“cow* milk*" OR "bovine* milk*" OR "goat* milk*" OR "caprine milk*" OR "buffalo* milk*" OR “camel* milk*" OR "sheep* milk*" OR "ovine milk*")
2 AB=(“cow* milk*" OR "bovine* milk*" OR "goat* milk*" OR "caprine milk*" OR "buffalo* milk*" "camel* milk*" OR "sheep* milk*" OR "ovine milk*")

3 #2 OR #1

4 TI=(formula*)

5 AB=(formula*)

6 #5 OR #4

7 TI=(infant* OR infancy OR baby OR babies OR neonat* OR "neo nat*" OR newborn* OR "new* born*" OR child* OR youth OR juvenile*)

8 AB=(infant* OR infancy OR baby OR babies OR neonat* OR "neo nat*" OR newborn* OR "new* born*" OR child* OR youth OR juvenile*)

9 #8 OR #7

10 #9 AND #6 AND #3

11 #9 AND #6 AND #3 Exclude Medline

LILACS

ti:("cow milk*" OR "cow's milk*" OR "cows milk*" OR "bovine milk*" OR "bovine's milk*" OR "goat milk*" OR "goat's milk*" OR "goats milk*" OR "caprine milk*" OR "buffalo milk*" OR "buffalo's milk*" OR "camel milk*" OR "camel’s milk*" OR "camels milk*" OR "sheep milk*" OR "sheep’s milk*" OR "ewe milk*" OR "ewe’s milk*" OR "ewes milk*" OR "ovine milk*") OR (ab:("cow milk*" OR "cow's milk*" OR "cows milk*" OR "bovine milk*" OR "bovine's milk*" OR "goat milk*" OR "goat's milk*" OR "goats milk*" OR "caprine milk*" OR "buffalo milk*" OR "buffalo's milk*" OR "camel milk*" OR "camel’s milk*" OR "camels milk*" OR "sheep milk*" OR "sheep’s milk*" OR "ewe milk*" OR "ewe’s milk*" OR "ewes milk*" OR "ovine milk*")) AND (((ti:(formula*)) OR (ab:(formula*))) OR ((mh:("infant formula")))) AND (((ti:(infant* OR infancy OR baby OR babies OR neonat* OR "neo nat*" OR newborn* OR "new born*" OR "newly born*" OR child* OR youth OR juvenile*)) OR (ab:(infant* OR infancy OR baby OR babies OR neonat* OR "neo nat*" OR newborn* OR "new born*" OR "newly born*" OR child* OR youth OR juvenile*))) OR ((mh:("infant")) OR ((mh:("child"))))) AND NOT ((mh:("animals")) AND NOT ((mh:("animals")) AND ((mh:("humans")))))

Global Index Medicus

ti:("cow milk*" OR "cow's milk*" OR "cows milk*" OR "bovine milk*" OR "bovine's milk*" OR "goat milk*" OR "goat's milk*" OR "goats milk*" OR "caprine milk*" OR "buffalo milk*" OR "buffalo's milk*" OR "camel milk*" OR "camel’s milk*" OR "camels milk*" OR "sheep milk*" OR "sheep’s milk*" OR "ewe milk*" OR "ewe’s milk*" OR "ewes milk*" OR "ovine milk*") OR (ab:("cow milk*" OR "cow's milk*" OR "cows milk*" OR "bovine milk*" OR "bovine's milk*" OR "goat milk*" OR "goat's milk*" OR "goats milk*" OR "caprine milk*" OR "buffalo milk*" OR "buffalo's milk*" OR "camel milk*" OR "camel’s milk*" OR "camels milk*" OR "sheep milk*" OR "sheep’s milk*" OR "ewe milk*" OR "ewe’s milk*" OR "ewes milk*" OR "ovine milk*")) AND (((ti:(formula*)) OR (ab:(formula*))) OR ((mh:("infant formula")))) AND (((ti:(infant* OR infancy OR baby OR babies OR neonat* OR "neo nat*" OR newborn* OR "new born*" OR "newly born*" OR child* OR youth OR juvenile*)) OR (ab:(infant* OR infancy OR baby OR babies OR neonat* OR "neo nat*" OR newborn* OR "new born*" OR "newly born*" OR child* OR youth OR juvenile*))) OR ((mh:("infant")) OR ((mh:("child"))))) AND NOT ((mh:("animals")) AND NOT ((mh:("animals")) AND ((mh:("humans")))))AND (db:("WPRIM" OR "IMEMR" OR "IMSEAR" OR "AIM"))

Table S1: List of publications of included studies

Title	Authors	Published Year	Merged Citation Title
Cow milk feeding in infancy: gastrointestinal blood loss and iron nutritional status.	Fomon SJ; Ziegler EE; Nelson SE; Edwards BB	1981	
Iron status and intake of older infants fed formula vs cow milk with cereal	Fuchs, G. J.; Farris, R. P.; DeWier, M.; Hutchinson, S. W.; Warrier, R.; Doucet, H.; Suskind, R. M.	1993	Fuchs G, DeWier M, Hutchinson S, Sundeen M, Schwartz S, Suskind R. Gastrointestinal blood loss in older infants: impact of cow milk versus formula. J Pediatr Gastroenterol Nutr. 1993 Jan;16(1):4-9<br><br>Fuchs GJ, Farris RP, DeWier M, et al. Iron status and intake of older infants fed formula vs cow milk with cereal. Am J Clin Nutr 1993;58:343-8.<br><br>Fuchs, G. J. Clemens, R. A. Hutchinson, S. W. et al, Growth of older infants fed low-fat formula: Nutrition Research, Volume 16, Issue 3, 1996, Pages 391-400,
Cow milk feeding in infancy: further observations on blood loss from the gastrointestinal tract	Ziegler, E. E.; Fomon, S. J.; Nelson, S. E.; Rebouche, C. J.; Edwards, B. B.; Rogers, R. R.; Lehman, L. J.	1990	
Consequences of starting whole cow milk at 6 months of age	Tunnessen, W. W., Jr.; Oski, F. A.	1987	
Iron fortified follow on formula from 9 to 18 months improves iron status but not development or growth: a randomised trial	Morley, R.; Abbott, R.; Fairweather-Tait, S.; MacFadyen, U.; Stephenson, T.; Lucas, A.	1999	
Iron status of one-year-olds and association with breast milk, cow's milk or formula in late infancy	Thorisdottir, A. V.; Ramel, A.; Palsson, G. I.; Tomassson, H.; Thorsdottir, I.	2013	

Prevalence of iron deficiency in 12-mo-old infants from 11 European areas and influence of dietary factors on iron status (Euro-Growth study)	Male, C.; Persson, L. A.; Freeman, V.; Guerra, A.; van't Hof, M. A.; Haschke, F.	2001	
Comparative Metabolic Study of Older Infants Fed Infant Formula, Transition Formula, or Whole Cows Milk	Fuchs, G. J.; Gastanaduy, A. S.; Suskind, R. M.	1992	
Effects on childhood body habitus of feeding large volumes of cow or formula milk compared with breastfeeding in the latter part of infancy	Hopkins, D.; Steer, C. D.; Northstone, K.; Emmett, P. M.	2015	

Figure S1: Effect of animal's milk vs formula milk intake in infants 6-11 month of age on Anemia: Subgroup analysis based on age of initiation

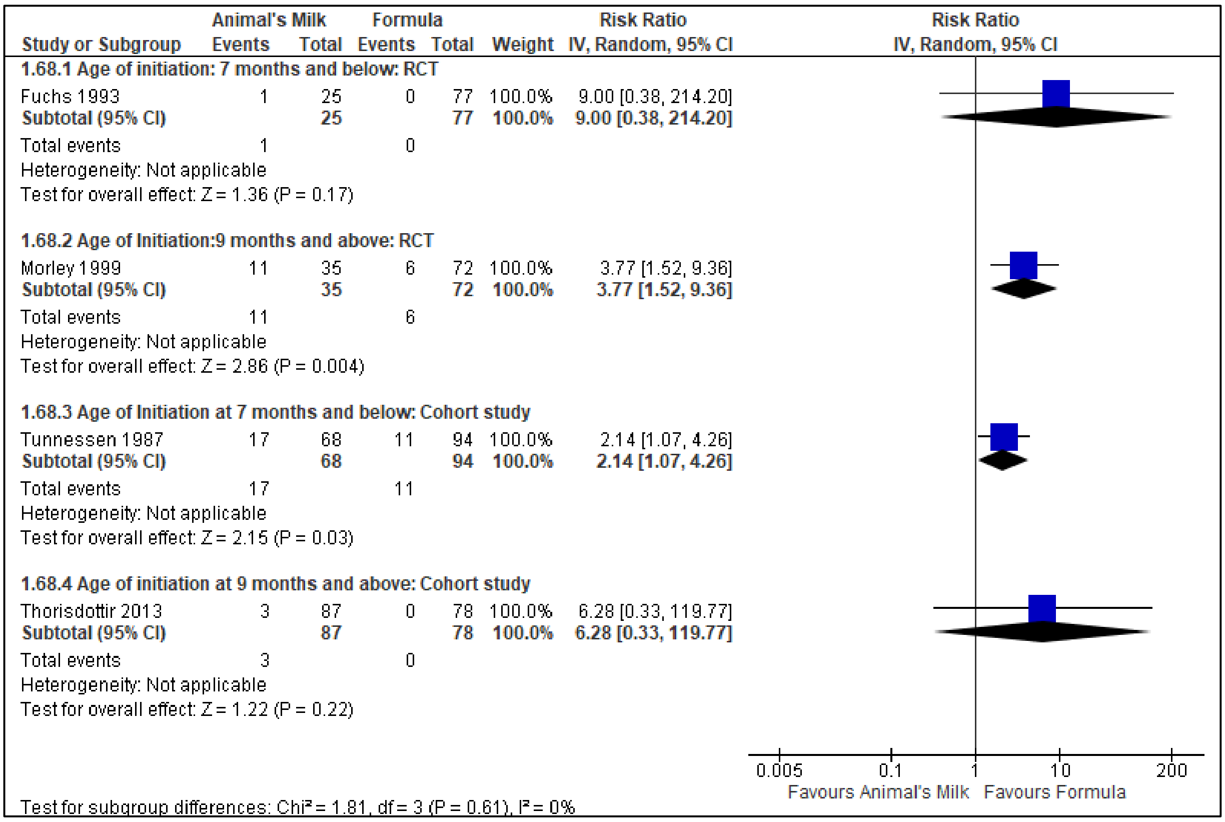

Figure S2: Effect of animal's milk vs formula milk intake in infants 6-11 month of age weight for age: Subgroup analysis based on age of initiation

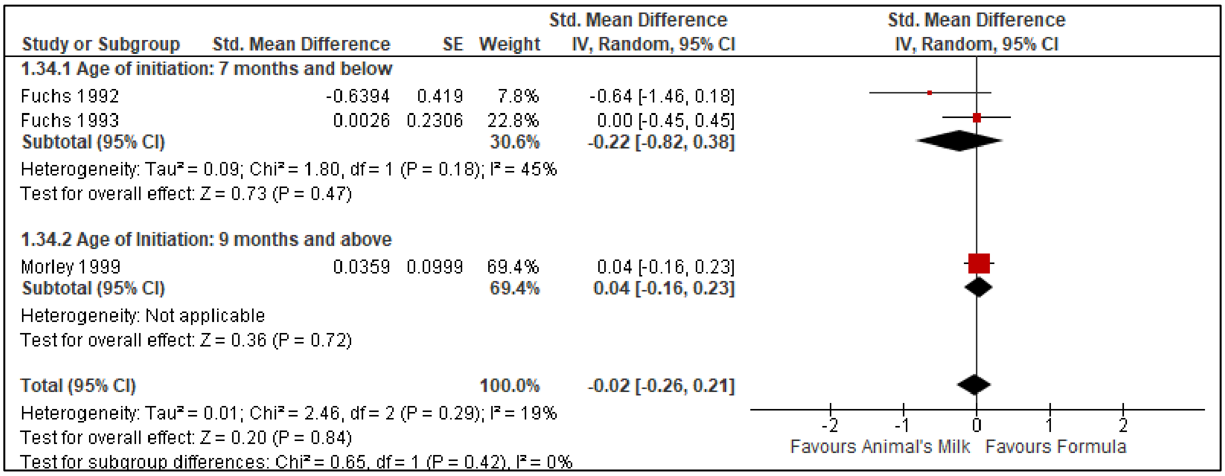

Table S2: List of excluded studies and reason for exclusion

Abrams 1997[1]	exclude wrong study design: looked at different mineral levels in infants fed just breast milk and solid foods. Does not compare formula or animal's milk.
AfeicheZehil 2017[2]	Excluded for wrong study design as data collection was a 24-hour recall of dietary intake rather than a longitudinal study.
Anderson 1987[3]	Full text was not available.
Balogun 1994[4]	Excluded for wrong study design. They are examining the elemental composition of different milks rather than using the milks in infants. There is no patient population.
Bano 2008[5]	Exclude for wrong study design as this is a descriptive and cross-sectional study on infants up to the age of 6 months being fed either mothers` milk, infant formula or cow/buffalo milk.
Borovik 2017[6]	Excluded for wrong intervention: comparing breastmilk to formula and looking at intervention starting from 0-5 months
Chessare 1988[7]	Excluded for wrong study design: not a study, just a reply to the editor reviewing the Tunnessen and Oski (J PEDIATR 1987;111:813-6) study which we are including
Davidsson 1989[8]	Excluded for wrong study population. The mean age was 28 years (range, 21 to 45 years). We are at looking infants aged 4-11 months.
Donovan 1988[9]	Excluded for wrong study design and wrong intervention as this is a basic science study looking at nutrient and protein differences between cow milk, goat milk and infant formula
Frey 1986[10]	Full text was not available.
Fuchs 1991[11]	Duplicate of study Fuchs 1991 (see below).
Fuchs 1991[12]	Duplicate: abstract of Fuchs 1992 study (included)
Gruskay 1982[13]	Excluded for wrong patient population: started intervention in non-breastfed infants at birth rather than at 4-11 months. Additionally, it is unclear if the milk formula is cow's milk or actually formula as there are no details on how the formula was made in which case it would also be excluded for wrong intervention.
Hachelaf 1993[14]	Excluded for wrong intervention as they are comparing goat’s milk to cow's milk rather than animal's milk to formula. Additionally, children are aged 9-72 months of age and the study does not break up results by age groups, so most of these children would be outside of our study range (4-11 months).
Hong-Seok 1997[15]	Full text was not available.
Host 1988[16]	Excluded for wrong patient population and wrong intervention. They are comparing homogenized milk vs non-homogenized milk allergic patients and the median age was 14 months.
Jury 1991[17]	Full text was not available.
Kersting 1988[18]	Full text was not available.
Knip 2018[19]	Excluded for wrong intervention as both groups were fed formula rather than comparing formula to cow's milk. Additionally, the outcomes were only based on diabetes status rather which is not related to our primary or secondary outcomes.
Lapointe 1952[20]	Full text was not available.
Laubereau 2004[21]	Excluded for wrong intervention: studying breastmilk vs milk-based formulas. Also, wrong age group, they started their study at birth and are looking at the first 2 weeks of life rather than at 4-11 months.
Lovell 2019[22]	Excluded for wrong patient population. They are starting the intervention at 12 months rather than at 4-11 months (our inclusion criteria)
Lucas 1990[23]	Excluded for wrong intervention as they are not looking at cow's milk.
MacLean 1978[24]	Excluded for wrong patient population as the mean age was 17 months rather than 4-11 months (our inclusion criteria). Additionally, they are studying cow's milk formula rather than raw animal's milk.

Magalhães 2012[25]	Excluded for wrong patient population as data collection for infant feeding is taken "in first months of life" and does not delineate between the ages. We are specifically looking at comparing formula to animal's milk feedings starting between the ages of 4-11 months of age.
Martin 2003[26]	Exclude for wrong study design as this was a long term follow up study and they were looking at milk supplementation rather than comparing cow's milk to formula
Mennella 2015[27]	exclude wrong intervention: they are not comparing to animals' milk but instead to formula and starting treatment at .5 months rather than at 4-11 months of age
Mennella 2016[28]	This is a duplicate of the above study (Mennella 2016)
Mills 1990[29]	Excluded for wrong study design. Infants were studied with a single screening survey of children aged 8-24 months.
Mimouni 1993[30]	Excluded for wrong intervention: comparing three formula groups: one not animal milk.
Montalto 1985[31]	Excluded for wrong outcomes as they are looking at protein, sodium, potassium, solid food intake, etc. of those fed breast, cow's milk or formula.
Naudé,1979[32]	Excluded for wrong intervention as this study is looking at soy formula vs. milk-based formula.
Nct 2006[33]	Duplicate of Nielsen 2007
Nielsen 2007[34]	Excluded for wrong outcomes: right population and intervention but they are only looking at the microbiota of the infants.
Ozkan 1994[35]	Excluding for wrong intervention as they are looking at formula vs breastfeeding.
Penrod 1988[36]	Excluded as "duplicate" as this is the abstract for Penrod 1990.
Penrod 1990[37]	Excluded for wrong study design: a cross-sectional study.
Penrod 1990[38]	Excluded: duplicate
Pirila 2011[39]	Excluded for wrong patient population as this study is a follow-up of the original cohort study and is focusing on adults rather than on children.
Qudsia 2015[40]	Excluded for wrong comparator. This study is looking at fortified milk fed vs cow's milk fed vs breast feeding. They are not comparing formula vs milk.
Radke 2017[41]	Excluded for wrong intervention: comparing two formulas (one with and one without probiotics) rather than looking at animal's milk
Rapetti 1997[42]	Excluded for wrong population: these infants are 12 - 48 months (our inclusion criteria are infants that started the intervention between ages 4-11 months). Additionally, these infants already have anemia/ iron deficiency.
Riikonen 2016[43]	Excluded for wrong study design as this study is a descriptive study looking at percent of people feeding what foods from birth - 1 year of life and what the first complementary feedings were
Rzehak 2009[44]	Excluded for wrong intervention (they are comparing formulas with partially hydrolyzed whey, extensively hydrolyzed whey, extensively hydrolyzed casein or cow-milk formula and infants exclusively breastfed).
Rzehak 2011[45]	Excluded for wrong intervention (they are comparing formulas with partially hydrolyzed whey, extensively hydrolyzed whey, extensively hydrolyzed casein or cow-milk formula and infants exclusively breastfed).
Rzehak 2013[46]	Excluded for wrong intervention: they were comparing formulas rather than animals milk vs formula
Saarinen 1977[47]	Excluded for wrong intervention as they are looking at cow's milk vs. breastmilk NOT formula and started treatment before 2 months rather than at 4-11 months
Saarinen 1979[48]	Exclude wrong comparator. Says "homemade cow's milk formula" but does not say how it was prepared. Additionally, intervention was started at 2 months rather than at 4-11 months of age.
Saarinen 1979[49]	Excluded wrong comparator. Definition of the formula did not qualify as formula. Only necrose was added to commercially available daily milk.
Saarinen 1979[50]	Excluded for wrong outcomes. We would only be looking at the BII group (short breastfeeding and then switched to cow's milk or formula at 2-6 months) but the paper does not separate results for the BII group into cow's milk vs formula.
Saarinen 1999[51]	Excluded: duplicate
Saarinen 2000[52]	Excluded for wrong intervention: studied cow's milk formula rather than raw cow's milk.
Sacri 2016[53]	Excluded for wrong study design: abstract to a cross-sectional study published a year later.
Sadowitz 1983[54]	Excluded for wrong study design. Only a single sample was taken (iron test) and dietary information was collected form each patient on when age of feeding began. There is no longitudinal component to this study.
Sakihara 2020[55]	Exclude for wrong intervention: they are looking at cow's milk formula.
Saldan 2017[56]	Exclude for wrong study design: cross sectional study.
Schmitz 1992[57]	Excluded for wrong intervention: comparing formula vs. formula.
Schrander 1993[58]	Excluding for wrong study design: cross-sectional study
Shank 1987[59]	Full text not found and based on the abstract this is likely a cross-sectional study.
Shank 1992[60]	Wrong study design: cross sectional study
Sobik 2020[61]	Excluded for wrong intervention: comparing milk-based formula to soy formula (rather than raw animal milk).
Southby 1948[62]	Full text was not available.
Specker 1991[63]	Excluded wrong intervention: studying breastmilk vs milk-based formulas.
Tannock 2013[64]	Excluded wrong intervention: comparing 2 types of formula (cow and goat).
Thomas 1986[65]	Excluded: duplicate of Thomas 1986 (see below)
Thomas 1986[66]	Excluded for wrong study design. This is a cross sectional study in which a single, randomly selected stool sample for determination of FA1AT was obtained from each subject.

Thorisdottir 2011[67]	Exclude for wrong outcomes, wrong study design and wrong intervention. This study does not compare formula to cow's milk. Instead, this is a series of surveys done at 0-4, 4-8 and 8-12 months to study nutrient intakes based on those surveys for the study population.
Ummarino 2003[68]	Excluded for wrong study design as they are looking at the education level of mothers who feed infants inappropriately rather than comparing formula to milk.
Venkataraman 1992[69]	Full text was not available.
Vesikari 1986[70]	Excluded for wrong outcomes as they are only looking at IgG and IgM antibodies after rotavirus vaccination.
Vesikari 1986[71]	Excluded as this is a duplicate of the above study (Vesikari 1986)
Virtanen 1998[72]	Excluded for wrong outcomes: looking at diabetes incidence after starting milk at various ages rather than comparing formula to milk drinking
vonBerg 2003[73]	Excluded for wrong intervention: they are studying three hydrolyzed formulas to a conventional cow's milk-based formula
VonBerg 2009[74]	Excluded: wrong intervention. They are looking at 3 hydrolyzed formulas were compared with standard CMF
Vossenaar 2015[75]	Excluded wrong study design: this is a questionnaire-based study on feeding preferences vs. demographics of infants.
Walter 1990[76]	Full text not available.
Wharton 1992[77]	Full text not available.
Woodruff 1972[78]	Exclude wrong study design: started cow's milk at 2 months of age rather than at 4-11 months of age (the group we are looking at)
Woodruff 1987[79]	Full text was not available.
Yagi 1986[80]	Excluded: wrong outcomes. This only compares the protein and growth factor levels in cow's milk compared to breastmilk and formulas. there is no treatment of infants involved.
Yeung 1982[81]	Excluded for wrong comparator as they are comparing 2% milk to formula + cow's milk together rather than comparing 2% milk to formula alone.
Zhou 2011[82]	Excluded for wrong intervention as both intervention and control are formulas
Ziegler 1999[83]	Excluding for wrong comparator as they are studying hemoglobin levels after all infants start cow's milk after a 2-month period in which they are fed formula. They are also comparing infants who were previously breastfed and then fed cow's milk to infants who are formula fed and start cow's milk feedings.
Zimring 1986[84]	Full text was not available.
COW'S MILK VERSUS HUMAN MILK PROTEIN IN INFANT FEEDING 1962[85]	Excluded for wrong study design: review

Table S3: Handling of data from individual studies for inclusion in the meta-analysis

Fomon 1981	Combined whole milk and heat-treated cow's milk for boys and girls
Fuchs 1993/1996	Combined the three formula groups (2 low fat follow-up formulas and a standard infant formula) and males/females
Morley 1999	Combined unfortified formula and iron fortified formula groups
Fuchs 1992	Combined follow up formula and standard infant formula. Added the increments and used data at 12 months of age. Used standard deviations from Maldanado 2010.

Table S4: Study definitions of outcome parameters of anemia and gastrointestinal blood loss

Study	Anemia definition	Gastrointestinal blood loss definition
Morley 1999	haemoglobin value below 110 g/ litre	n/a
Fuchs 1993	Hb < 105 g/L	n/a
Thorisdottir 2013	The cutoff points used for IDA (iron deficiency anemia) were Hb <105 g/l, MCV < 74 fl and SF < 12 lg/l	n/a
Tunnessen 1987	Hemoglobin (g/dL) < 11	n/a
Fomon 1981	n/a	Guaic-positive stools (at 196 days)
Ziegler 1990	n/a	Guaic-positive stools (at 252 days)

Abbreviations: n/a not available

References

1. Abrams, S.A.; Wen, J.; Stuff, J.E. Absorption of calcium, zinc, and iron from breast milk by five- to seven-month-old infants. *Pediatr Res* **1997**, *41*, 384-390, doi:10.1203/00006450-199703000-00014.
2. Afeiche Zehil, M.; Eldridge, A.; Fries, L.R.; Reidy, K.; Villalpando, S. Recommended and inappropriate beverage intake in Mexican infants and toddlers. *Annals of Nutrition and Metabolism* **2017**, *71*, 613-614, doi:10.1159/000480486.
3. Anderson, K.; Frey, D.; Acosta, P.B. Cows Milk or Milk-Based Formula - Manganese Intake and Plasma-Levels. *Federation Proceedings* **1987**, *46*, 1192-1192.
4. Balogun, F.A.; Akanle, O.A.; Spyrou, N.M.; Owa, J.A. A Comparative-Study of Elemental Composition of Human Breast-Milk and Infant Milk Substitutes. *Biological Trace Element Research* **1994**, *43-5*, 471-479, doi:10.1007/BF02917349.
5. Bano, N.; Jabbar, A.; Mustansar, M.; Ch, Z.A. Comparison of potential renal solute load (prsl) in healthy breastfed and non breastfed infants. *Esculapio j services inst med sci* **2008**, *3*, 14-20.
6. Borovik, Ò.E.; Semyonova, N.N.; Lukoyanova, O.L.; Zvonkova, N.G.; Bushueva, T.V.; Stepanova, T.N.; Skvortsova, V.A.; Melnichuk, O.S.; Kopyltsova, E.A.; Semikina, E.L.; et al. Efficiency of using the adapted goat's milk formula in the diet of healthy young infants: A multicenter prospective comparative study. *Voprosy Sovremennoi Pediatrii - Current Pediatrics* **2017**, *16*, 226-234, doi:10.15690/vsp.v16i3.1733.
7. Chessare, J.B. Whole cow milk versus iron-fortified formula. *J Pediatr* **1988**, *112*, 1049-1050, doi:10.1016/s0022-3476(88)80251-8.
8. Davidsson, L.; Cederblad, A.; Lonnerdal, B.; Sandstrom, B. Manganese Absorption from Human-Milk, Cows Milk, and Infant Formulas in Humans. *American Journal of Diseases of Children* **1989**, *143*, 823-827, doi:10.1001/archpedi.1989.02150190073024.
9. Donovan, S.M.; Kunz, C.; Rudloff, S.; Lonnerdal, B. Characterization of Soluble and Insoluble Proteins in Human-Milk, Cow Milk, Goat Milk and Infant Formulas. *Faseb Journal* **1988**, *2*, A651-A651.
10. Frey, D.; Anderson, K.; Acosta, P.B. Copper and Zinc Status of Infants Fed Either Cow Milk or Milk Based Formula. *Federation Proceedings* **1986**, *45*, 589-589.
11. Fuchs, G.J.; Dewier, M.; Hutchinson, S.W.; Farris, R.P.; Doucet, H.; Schwartz, S.; Suskind, R.M. Impact of Whole Cows Milk with Iron-Fortified Cereal or Formula on Dietary-Intake and Iron Status of Infants 6-12 Months of Age. *Clinical Research* **1991**, *39*, A646-A646.
12. Fuchs, G.J.; DeWier, M.; Hutchinson, S.W.; Farris, R.P.; Doucet, H.; Schwartz, S.; Suskind, R.M. Impact of whole cow's milk with iron-fortified cereal or formula on dietary intake and iron status of infants 6-12 months of age. *American journal of clinical nutrition* **1991**, *53*, P-18.

13. Gruskay, F.L. Comparison of breast, cow, and soy feedings in the prevention of onset of allergic disease: a 15-year prospective study. *Clin Pediatr (Phila)* **1982**, *21*, 486-491, doi:10.1177/000992288202100807.
14. Hachelaf, W.; Boukhrelda, M.; Benbouabdellah, M.; Coquin, P.; Desjeux, J.F.; Boudraa, G.; Touhami, M. Comparative Digestibility of Goats Versus Cows Milk Fats in Children with Digestive Malnutrition. *Lait* **1993**, *73*, 593-599.
15. Hong-Seok, A.H.N.; hyun-Sook, B.A.I.; Sung-Hye, P.; Eun-jung, C.; Kyung-Hwan, O.H. Serum Concentration of Major Minerals, Trace Elements, Lipids and Fatty Acids Composition Related to Whole Cow's Milk Feeding in Infancy & Young Childhood. *Korean Journal of Community Nutrition* **1997**, 477-485.
16. Host, A.; Samuelsson, E.G. Allergic Reactions to Raw, Pasteurized, and Homogenized Pasteurized Cow Milk - a Comparison - a Double-Blind Placebo-Controlled Study in Milk Allergic Children. *Allergy* **1988**, *43*, 113-118, doi:10.1111/j.1398-9995.1988.tb00404.x.
17. Jury, G.; Castillo, C.; Atalah, E.; Puentes, R.; Riumallo, J. [Growth, acceptance, and tolerance with a new milk formula]. *Rev Chil Pediatr* **1991**, *62*, 87-93.
18. Kersting, M.; Chahda, C.; Schoch, G. Alternative Diets for Infants - Comparative-Study .1. Milk Mixtures for Infants in the 1st Months of Life. *Ernahrungs-Umschau* **1988**, *35*, 203-&.
19. Knip, M.; Åkerblom, H.K.; Al Taji, E.; Becker, D.; Bruining, J.; Castano, L.; Danne, T.; de Beaufort, C.; Dosch, H.M.; Dupre, J.; et al. Effect of Hydrolyzed Infant Formula vs Conventional Formula on Risk of Type 1 Diabetes: The TRIGR Randomized Clinical Trial. *Jama* **2018**, *319*, 38-48, doi:10.1001/jama.2017.19826.
20. Lapointe, D.; Dechene, E.; Larve, A. [Comparative clinical study of two milk formulas; evaporated milk with dextri-maltose; cow's milk with sugar]. *Laval Med* **1952**, *17*, 618-624.
21. Laubereau, B.; Brockow, I.; Zirngibl, A.; Koletzko, S.; Gruebl, A.; von Berg, A.; Filipiak-Pittroff, B.; Berdel, D.; Bauer, C.P.; Reinhardt, D.; et al. Effect of breast-feeding on the development of atopic dermatitis during the first 3 years of life--results from the GINI-birth cohort study. *Journal of pediatrics* **2004**, *144*, 602-607.
22. Lovell, A.L.; Milne, T.; Jiang, Y.; Chen, R.X.; Grant, C.C.; Wall, C.R. Evaluation of the Effect of a Growing up Milk Lite vs. Cow's Milk on Diet Quality and Dietary Intakes in Early Childhood: The Growing up Milk Lite (GUMLi) Randomised Controlled Trial. *Nutrients* **2019**, *11*, doi:10.3390/nu11010203.
23. Lucas, A.; Brooke, O.G.; Morley, R.; Cole, T.J.; Bamford, M.F. Early Diet of Preterm Infants and Development of Allergic or Atopic Disease - Randomized Prospective-Study. *British Medical Journal* **1990**, *300*, 837-840, doi:10.1136/bmj.300.6728.837.
24. MacLean, W.C., Jr.; Placko, R.P.; Graham, G.G. Fasting plasma free amino acids of infants and children consuming cow milk proteins. *Johns Hopkins Med J* **1978**, *142*, 147-151.
25. Magalhães, T.C.; Vieira, S.A.; Priore, S.E.; Ribeiro, A.Q.; Lamounier, J.A.; Franceschini, S.C.; Sant'Ana, L.F. Exclusive breastfeeding and other foods in the first six months of life: effects on nutritional status and body composition of Brazilian children. *ScientificWorldJournal* **2012**, *2012*, 468581, doi:10.1100/2012/468581.

26. Martin, R.M.; McCarthy, A.; Smith, G.D.; Davies, D.P.; Ben-Shlomo, Y. Infant nutrition and blood pressure in early adulthood: the Barry Caerphilly Growth study. *Am J Clin Nutr* **2003**, *77*, 1489-1497, doi:10.1093/ajcn/77.6.1489.
27. Mennella, J.; Trabulsi, J.; Papas, M. Effects of cow milk versus extensive protein hydrolysate formulas on infant cognitive development. *FASEB Journal* **2015**, *29*.
28. Mennella, J.A.; Trabulsi, J.C.; Papas, M.A. Effects of cow milk versus extensive protein hydrolysate formulas on infant cognitive development. *Amino Acids* **2016**, *48*, 697-705, doi:10.1007/s00726-015-2118-7.
29. Mills, A.F. Surveillance for anaemia: risk factors in patterns of milk intake. *Arch Dis Child* **1990**, *65*, 428-431, doi:10.1136/adc.65.4.428.
30. Mimouni, F.; Campaigne, B.; Neylan, M.; Tsang, R.C. Bone Mineralization in the 1st Year of Life in Infants Fed Human-Milk, Cow-Milk Formula, or Soy-Based Formula. *Journal of Pediatrics* **1993**, *122*, 348-354, doi:10.1016/S0022-3476(05)83415-8.
31. Montalto, M.B.; Benson, J.D.; Martinez, G.A. Nutrient intakes of formula-fed infants and infants fed cow's milk. *Pediatrics* **1985**, *75*, 343-351.
32. Naudé, S.P.; Prinsloo, J.G.; Haupt, C.E. Comparison between a humanized cow's milk and a soy product for premature infants. *S Afr Med J* **1979**, *55*, 982-986.
33. Nct. Milk Types and Fish Oil in 9- to 12-Month-Old Infants. *https://clinicaltrials.gov/show/NCT00379171* **2006**.
34. Nielsen, S.; Nielsen, D.S.; Lauritzen, L.; Jakobsen, M.; Michaelsen, K.F. Impact of diet on the intestinal microbiota in 10-month-old infants. *J Pediatr Gastroenterol Nutr* **2007**, *44*, 613-618, doi:10.1097/MPG.0b013e3180406a11.
35. Ozkan, H.; Oren, H.; Erdag, N.; Cevik, N. Breast milk versus infant formulas: effects on intestinal blood flow in neonates. *Indian J Pediatr* **1994**, *61*, 703-709, doi:10.1007/bf02751984.
36. Penrod, J.C.; Acosta, P.B. Iron Status of Older Infants Fed Either Cows Milk or Milk Based Formula. *Journal of the American College of Nutrition* **1988**, *7*, 410-410.
37. Penrod, J.C.; Anderson, K.; Acosta, P.B. Impact on Iron Status of Introducing Cows Milk in the 2nd 6 Months of Life. *Journal of Pediatric Gastroenterology and Nutrition* **1990**, *10*, 462-467, doi:10.1097/00005176-199005000-00008.
38. Penrod, J.C.; Anderson, K.; Acosta, P.B. Impact on iron status of introducing cow's milk in the second six months of life. *J Pediatr Gastroenterol Nutr* **1990**, *10*, 462-467, doi:10.1097/00005176-199005000-00008.
39. Pirila, S.; Taskinen, M.; Viljakainen, H.; Kajosaari, M.; Turanlahti, M.; Saarinen-Pihkala, U.M.; Makitie, O. Infant Milk Feeding Influences Adult Bone Health: A Prospective Study from Birth to 32 Years. *Plos One* **2011**, *6*, doi:10.1371/journal.pone.0019068.
40. Qudsia, F.; Saboor, M.; Khosa, S.M.; Ayub, Q.; Moinuddin. Comparative analysis of serum iron, serum ferritin and red cell folate levels among breast fed, fortified milk and cow’s milk fed infants. *Pakistan Journal of Medical Sciences* **2015**, *31*, 706-709, doi:10.12669/pjms.313.6937.
41. Radke, M.; Picaud, J.C.; Loui, A.; Cambonie, G.; Faas, D.; Lafeber, H.N.; de Groot, N.; Pecquet, S.S.; Steenhout, P.G.; Hascoet, J.M. Starter formula enriched in prebiotics and probiotics ensures normal growth of infants and promotes gut health: a randomized clinical trial. *Pediatr Res* **2017**, *81*, 622-631, doi:10.1038/pr.2016.270.

42. Rapetti, M.C.; Donato, H.; deGalvagni, A.; Lubovitsky, M.; Lanzilotta, M.; Trepacka, E.; Burlando, G.; Weill, R. Correction of iron deficiency with an iron-fortified fluid whole cow's milk in children: Results of a pilot study. *Journal of Pediatric Hematology Oncology* **1997**, *19*, 192-196, doi:10.1097/00043426-199705000-00003.
43. Riikonen, A.; Hadley, D.; Uusitalo, U.; Miller, N.; Koletzko, S.; Yang, J.; Aronsson, C.A.; Hummel, S.; Norris, J.M.; Virtanen, S.M. Milk feeding and first complementary foods during the first year of life in an international prospective TEDDY cohort study. *Journal of Pediatric Gastroenterology and Nutrition* **2016**, *62*, 877-878, doi:10.1097/01.mpg.0000484500.48517.e7.
44. Rzehak, P.; Sausenthaler, S.; Koletzko, S.; Reinhardt, D.; von Berg, A.; Krämer, U.; Berdel, D.; Bollrath, C.; Grübl, A.; Bauer, C.P.; et al. Short- and long-term effects of feeding hydrolyzed protein infant formulas on growth at < or = 6 y of age: results from the German Infant Nutritional Intervention Study. *Am J Clin Nutr* **2009**, *89*, 1846-1856, doi:10.3945/ajcn.2008.27373.
45. Rzehak, P.; Sausenthaler, S.; Koletzko, S.; Reinhardt, D.; von Berg, A.; Kraemer, U.; Berdel, D.; Bollrath, C.; Gruebl, A.; Bauer, C.P.; et al. Long-term effects of hydrolyzed protein infant formulas on growth-extended follow-up to 10 y of age: results from the German Infant Nutritional Intervention (GINI) study. *American Journal of Clinical Nutrition* **2011**, *94*, 1803S-1807S, doi:10.3945/ajcn.110.000679.
46. Rzehak, P.; Grote, V.; Lattka, E.; Weber, M.; Gruszfeld, D.; Socha, P.; Closa-Monasterolo, R.; Escribano, J.; Giovannini, M.; Verduci, E.; et al. Associations of IGF-1 gene variants and milk protein intake with IGF-I concentrations in infants at age 6 months - results from a randomized clinical trial. *Growth Horm IGF Res* **2013**, *23*, 149-158, doi:10.1016/j.ghir.2013.05.002.
47. Saarinen, U.M.; Siimes, M.A.; Dallman, P.R. Iron absorption in infants: high bioavailability of breast milk iron as indicated by the extrinsic tag method of iron absorption and by the concentration of serum ferritin. *J Pediatr* **1977**, *91*, 36-39, doi:10.1016/s0022-3476(77)80439-3.
48. Saarinen, U.M.; Siimes, M.A. Iron absorption from breast milk, cow's milk, and iron-supplemented formula: an opportunistic use of changes in total body iron determined by hemoglobin, ferritin, and body weight in 132 infants. *Pediatr Res* **1979**, *13*, 143-147, doi:10.1203/00006450-197903000-00001.
49. Saarinen, U.M.; Siimes, M.A. Role of prolonged breast feeding in infant growth. *Acta Paediatr Scand* **1979**, *68*, 245-250, doi:10.1111/j.1651-2227.1979.tb04996.x.
50. Saarinen, U.M.; Kajosaari, M.; Backman, A.; Siimes, M.A. Prolonged breast-feeding as prophylaxis for atopic disease. *Lancet* **1979**, *2*, 163-166, doi:10.1016/s0140-6736(79)91432-6.
51. Saarinen, K.M.; Juntunen-Backman, K.; Jarvenpaa, A.L.; Kuitunen, P.; Lope, L.; Renlund, M.; Siivola, M.; Savilahti, E. Supplementary feeding in maternity hospitals and the risk of cow's milk allergy: A prospective study of 6209 infants. *Journal of Allergy and Clinical Immunology* **1999**, *104*, 457-461, doi:10.1016/S0091-6749(99)70393-3.
52. Saarinen, K.M.; Savilahti, E. Infant feeding patterns affect the subsequent immunological features in cow's milk allergy. *Clin Exp Allergy* **2000**, *30*, 400-406, doi:10.1046/j.1365-2222.2000.00732.x.

53. Sacri, A.S.; Hercberg, S.; Bocquet, A.; Vinatier, I.; Levy, C.; Blondel, B.; Vincelet, C.; Hebel, P.; De Montalembert, M.; Gouya, L.; et al. Iron deficiency prevalence and risk factors in children younger than 6 years-old in France: A population-based study. *Haematologica* **2016**, *101*, 606-607.
54. Sadowitz, P.D.; Oski, F.A. Iron status and infant feeding practices in an urban ambulatory center. *Pediatrics* **1983**, *72*, 33-36.
55. Sakihara, T.; Otsuji, K.; Arakaki, Y.; Hamada, K.; Sugiura, S.; Ito, K. Randomized trial of early infant formula introduction to prevent cow's milk allergy. *J Allergy Clin Immunol* **2020**, doi:10.1016/j.jaci.2020.08.021.
56. Saldan, P.C.; Venancio, S.I.; Saldiva, S.; Vieira, D.G.; Mello, D.F. MILK CONSUMPTION IN INFANTS UNDER ONE YEAR OF AGE AND VARIABLES ASSOCIATED WITH NON-MATERNAL MILK CONSUMPTION. *Rev Paul Pediatr* **2017**, *35*, 407-414, doi:10.1590/1984-0462/;2017;35;4;00004.
57. Schmitz, J.; Digeon, B.; Chastang, C.; Dupouy, D.; Leroux, B.; Robillard, P.; Strobel, S. Effects of brief early exposure to partially hydrolyzed and whole cow milk proteins. *J Pediatr* **1992**, *121*, S85-89, doi:10.1016/s0022-3476(05)81413-1.
58. Schrander, J.J.; van den Bogart, J.P.; Forget, P.P.; Schrander-Stumpel, C.T.; Kuijten, R.H.; Kester, A.D. Cow's milk protein intolerance in infants under 1 year of age: a prospective epidemiological study. *Eur J Pediatr* **1993**, *152*, 640-644, doi:10.1007/bf01955238.
59. Shank, J.S.; Dorsey, J.L.; Cooper, W.T.; Acosta, P.B. The Vitamin-E Status of Infants Receiving Cows Milk or Milk-Based Formula. *Federation Proceedings* **1987**, *46*, 1194-1194.
60. Shank, J.S.; Dorsey, J.L.; Anderson, K.; Cooper, W.T.; Acosta, P.B. Plasma vitamin E concentrations of older infants fed cow's milk or infant formula. *J Pediatr Gastroenterol Nutr* **1992**, *15*, 375-381, doi:10.1097/00005176-199211000-00003.
61. Sobik, S.; Sims, C.R.; McCorkle, G.; Bellando, J.; Sorensen, S.T.; Badger, T.M.; Casey, P.H.; Keith Williams, D.; Andres, A. Early infant feeding effect on growth and body composition during the first 6 years and neurodevelopment at age 72 months. *Pediatr Res* **2020**, doi:10.1038/s41390-020-01157-z.
62. Southby, R. Modification of cow's milk for infant feeding. *Med J Aust* **1948**, *1*, 660.
63. Specker, B.L.; Tsang, R.C.; Ho, M.L.; Landi, T.M.; Gratton, T.L. Low serum calcium and high parathyroid hormone levels in neonates fed 'humanized' cow's milk-based formula. *Am J Dis Child* **1991**, *145*, 941-945, doi:10.1001/archpedi.1991.02160080119033.
64. Tannock, G.W.; Lawley, B.; Munro, K.; Gowri Pathmanathan, S.; Zhou, S.J.; Makrides, M.; Gibson, R.A.; Sullivan, T.; Prosser, C.G.; Lowry, D.; et al. Comparison of the compositions of the stool microbiotas of infants fed goat milk formula, cow milk-based formula, or breast milk. *Appl Environ Microbiol* **2013**, *79*, 3040-3048, doi:10.1128/aem.03910-12.
65. Thomas, D.W.; McGilligan, K.M.; Carlson, M.; Azen, S.P.; Eisenberg, L.D.; Lieberman, H.M.; Rissman, E.M. Fecal Alpha-1-Antitrypsin and Hemoglobin Excretion in Healthy-Human Milk Milk-Fed, Formula-Milk-Fed, or Cows Milk-Fed Infants. *Pediatrics* **1986**, *78*, 305-312.
66. Thomas, D.W.; McGilligan, K.M.; Carlson, M.; Azen, S.P.; Eisenberg, L.D.; Lieberman, H.M.; Rissman, E.M. Fecal alpha 1-antitrypsin and hemoglobin excretion in healthy human milk-, formula-, or cow's milk-fed infants. *Pediatrics* **1986**, *78*, 305-312.

67. Thorisdottir, A.V.; Thorsdottir, I.; Palsson, G.I. Nutrition and Iron Status of 1-Year Olds following a Revision in Infant Dietary Recommendations. *Anemia* **2011**, *2011*, 986303, doi:10.1155/2011/986303.
68. Ummarino, M.; Albano, F.; De Marco, G.; Mangani, S.; Aceto, B.; Ummarino, D.; Correra, A.; Giannetti, E.; De Vizia, B.; Guarino, A. Short duration of breastfeeding and early introduction of cow's milk as a result of mothers' low level of education. *Acta Paediatr Suppl* **2003**, *91*, 12-17, doi:10.1111/j.1651-2227.2003.tb00641.x.
69. Venkataraman, P.S.; Neylan, M.J.; Carlson, J.; Setchell, K.D. Urinary Phytoestrogen Excretion in Infants - Differences between Human-Milk, Cow Milk Based, and Soy Based Formula Fed Infants. *Clinical Research* **1992**, *40*, A793-A793.
70. Vesikari, T.; Ruuska, T.; Delem, A.; Andre, F.E. Oral rotavirus vaccination in breast- and bottle-fed infants aged 6 to 12 months. *Acta paediatrica scandinavica* **1986**, *75*, 573-578.
71. Vesikari, T.; Ruuska, T.; Delem, A.; Andre, F.E. Oral Rotavirus Vaccination in Breast-Fed and Bottle-Fed Infants Aged 6 to 12 Months. *Acta Paediatrica Scandinavica* **1986**, *75*, 573-578, doi:10.1111/j.1651-2227.1986.tb10253.x.
72. Virtanen, S.M.; Hyppönen, E.; Läärä, E.; Vähäsalo, P.; Kulmala, P.; Savola, K.; Räsänen, L.; Aro, A.; Knip, M.; Akerblom, H.K. Cow's milk consumption, disease-associated autoantibodies and type 1 diabetes mellitus: a follow-up study in siblings of diabetic children. Childhood Diabetes in Finland Study Group. *Diabet Med* **1998**, *15*, 730-738, doi:10.1002/(sici)1096-9136(199809)15:9<730::Aid-dia646>3.0.Co;2-c.
73. von Berg, A.; Koletzko, S.; Grübl, A.; Filipiak-Pittroff, B.; Wichmann, H.E.; Bauer, C.P.; Reinhardt, D.; Berdel, D. The effect of hydrolyzed cow's milk formula for allergy prevention in the first year of life: the German Infant Nutritional Intervention Study, a randomized double-blind trial. *J Allergy Clin Immunol* **2003**, *111*, 533-540, doi:10.1067/mai.2003.101.
74. Von Berg, A.; Krämer, U.; Link, E.; Heinrich, J.; Brockow, I.; Koletzko, S.; Grübl, A.; Filipiak-Pittroff, B.; Wichmann, H.; Bauer, C.; et al. Childhood eczema: Intervention with hydrolysed infant formulas versus natural course. data from the giniplus study until the age of 6 years. *Allergy: European Journal of Allergy and Clinical Immunology* **2009**, *64*, 63-64, doi:10.1111/j.1398-9995.2009.02074.x.
75. Vossenaar, M.; Alvey, J.; van Beusekom, I.; Doak, C.M.; Solomons, N.W. Energy contribution from non-breastmilk items in low-income Guatemalan infants in their sixth month of life. *Salud Publica Mex* **2015**, *57*, 117-127, doi:10.21149/spm.v57i2.7407.
76. Walter, T.; Hertrampf, E.; Arredondo, M.; Vega, V. Effect of Different Milk Diets on Gastrointestinal Blood-Loss in Infancy. *Pediatric Research* **1990**, *28*, 296-296, doi:10.1203/00006450-199009000-00139.
77. Wharton, B. Which Milk for Normal Infants. *European Journal of Clinical Nutrition* **1992**, *46*, S27-S32.
78. Woodruff, C.W.; Wright, S.W.; Wright, R.P. The role of fresh cow's milk in iron deficiency. II. Comparison of fresh cow's milk with a prepared formula. *Am J Dis Child* **1972**, *124*, 26-30, doi:10.1001/archpedi.1972.02110130028004.
79. Woodruff, S.; Dorsey, J.; Nivens, D.; Acosta, P.B. Essential Fatty-Acid Status of Infants Fed Cows Milk or Milk Based Formula. *Federation Proceedings* **1987**, *46*, 1194-1194.

80. Yagi, H.; Suzuki, S.; Noji, T.; Nagashima, K.; Kuroume, T. Epidermal Growth-Factor in Cows Milk and Milk Formulas. *Acta Paediatrica Scandinavica* **1986**, *75*, 233-235, doi:10.1111/j.1651-2227.1986.tb10190.x.
81. Yeung, D.L.; Pennell, M.D.; Leung, M.; Hall, J. The effects of 2% milk intake on infant nutrition. *Nutrition Research* **1982**, *2*, 651-660, doi:10.1016/S0271-5317(82)80109-7.
82. Zhou, S.J.; Sullivan, T.; Gibson, R.A.; Makrides, M. How does goat milk infant formula compare to cow milk formula? A randomised controlled trial. *Journal of Pediatric Gastroenterology and Nutrition* **2011**, *52*, E208-E209, doi:10.1097/MPG.0b013e318224e326.
83. Ziegler, E.E.; Jiang, T.; Romero, E.; Vinco, A.; Frantz, J.A.; Nelson, S.E. Cow's milk and intestinal blood loss in late infancy. *J Pediatr* **1999**, *135*, 720-726, doi:10.1016/s0022-3476(99)70091-0.
84. Zimring, L.; Anderson, K.; Acosta, P.B. Protein Intakes of Infants Fed Either Cows Milk or Milk Based Formula. *Federation Proceedings* **1986**, *45*, 363-363.
85. COW'S MILK VERSUS HUMAN MILK PROTEIN IN INFANT FEEDING. *Nutrition Reviews* **1962**, *20*, 67-69, doi:10.1111/j.1753-4887.1962.tb04551.x.
